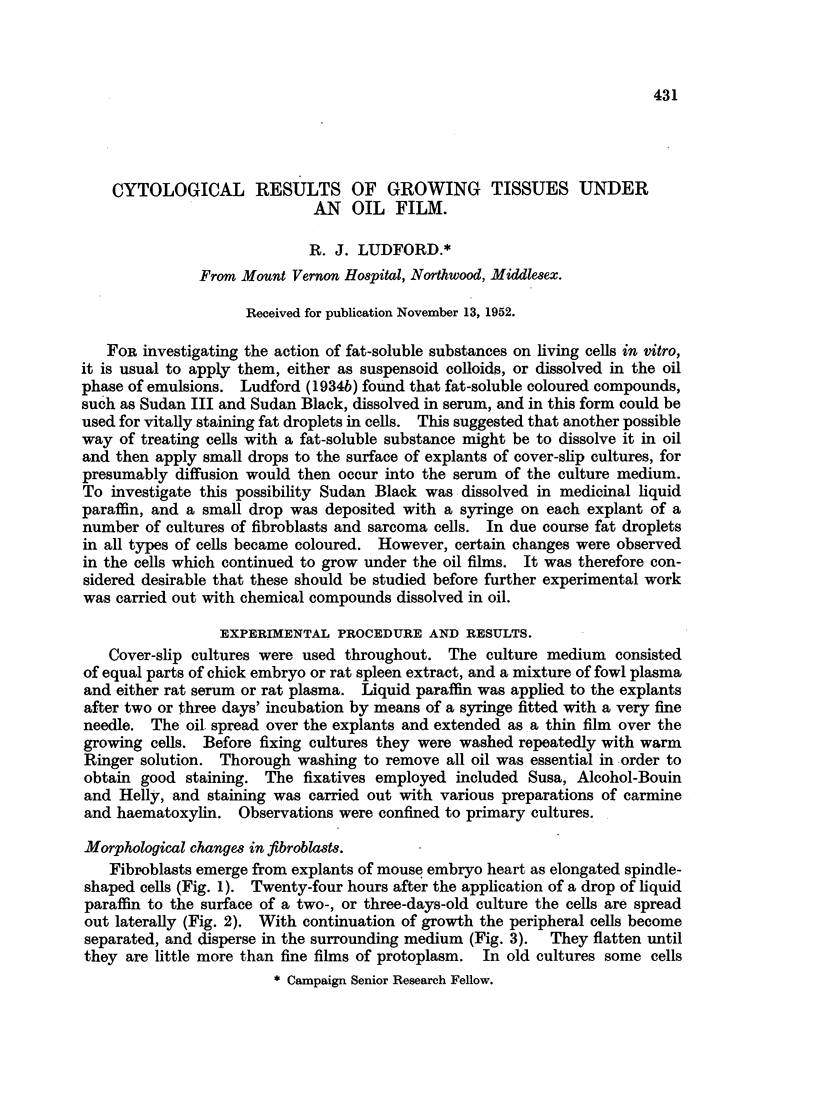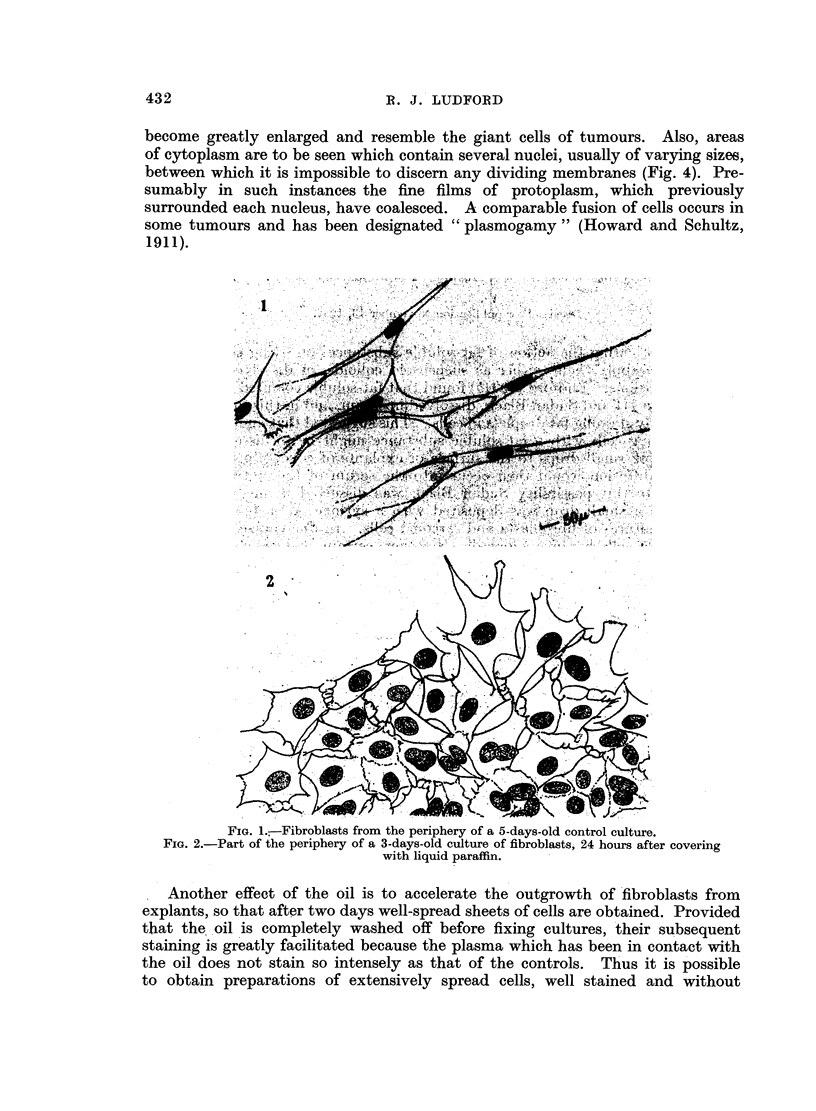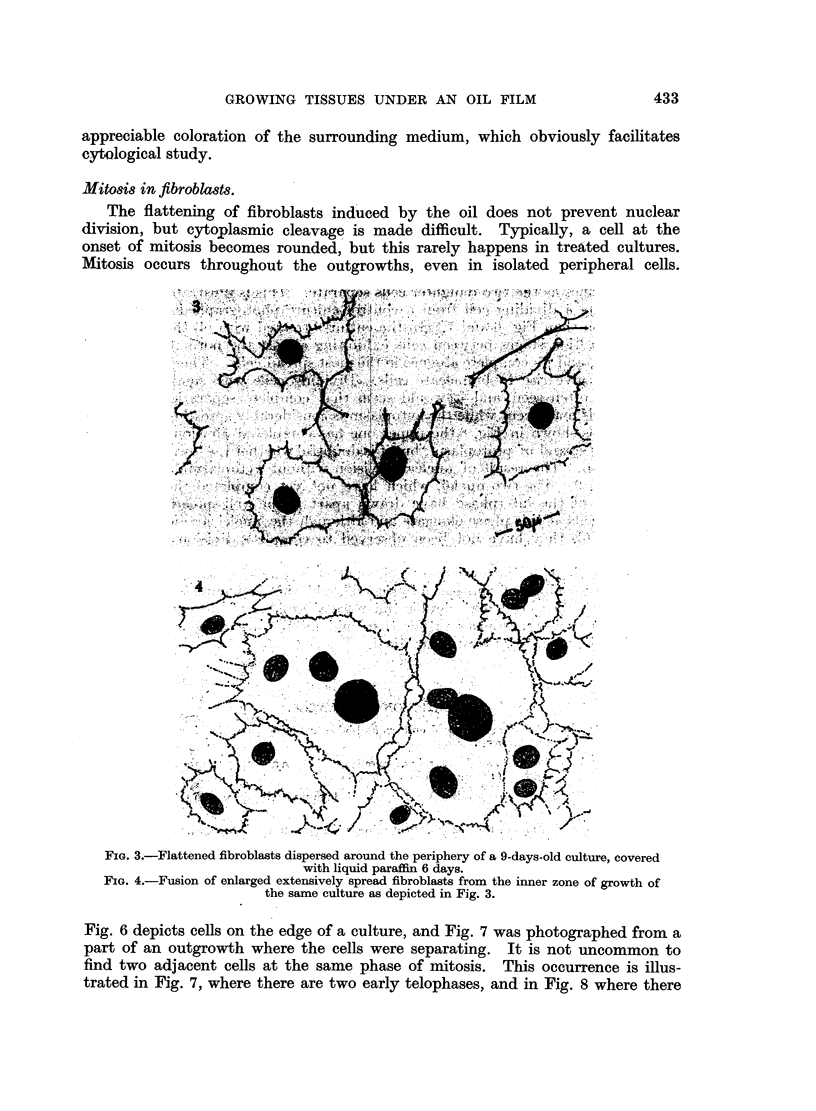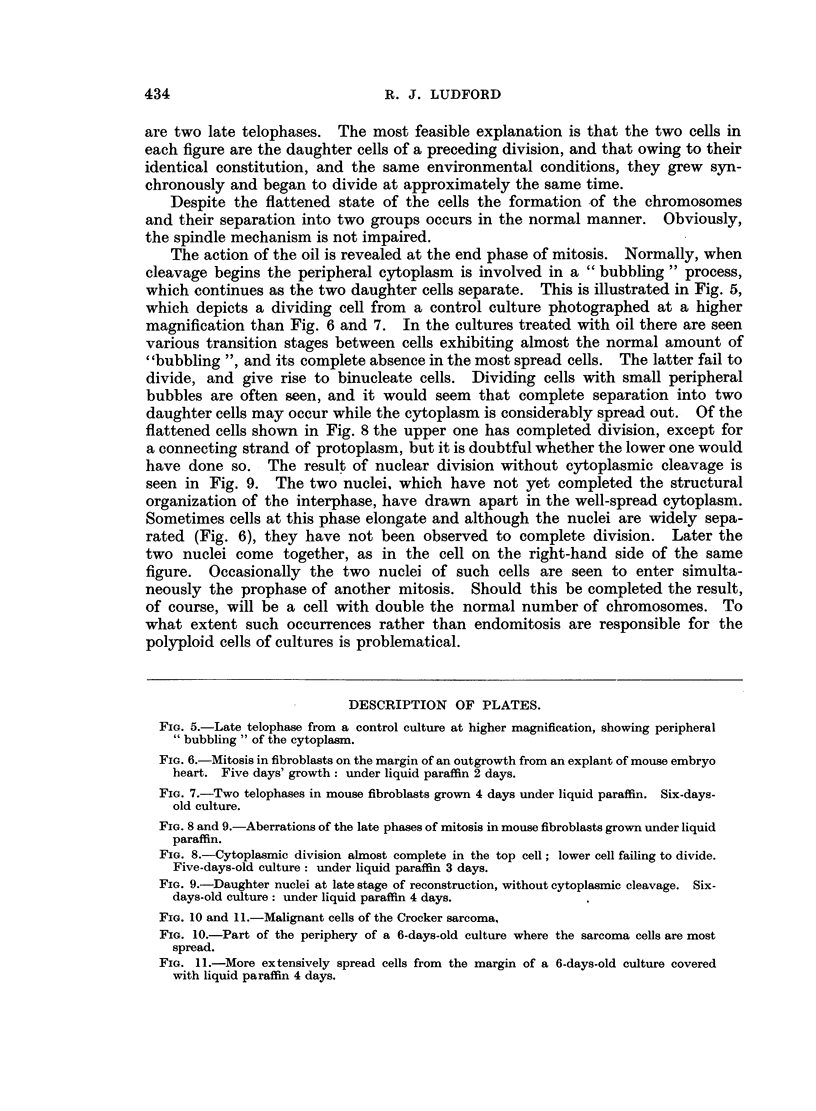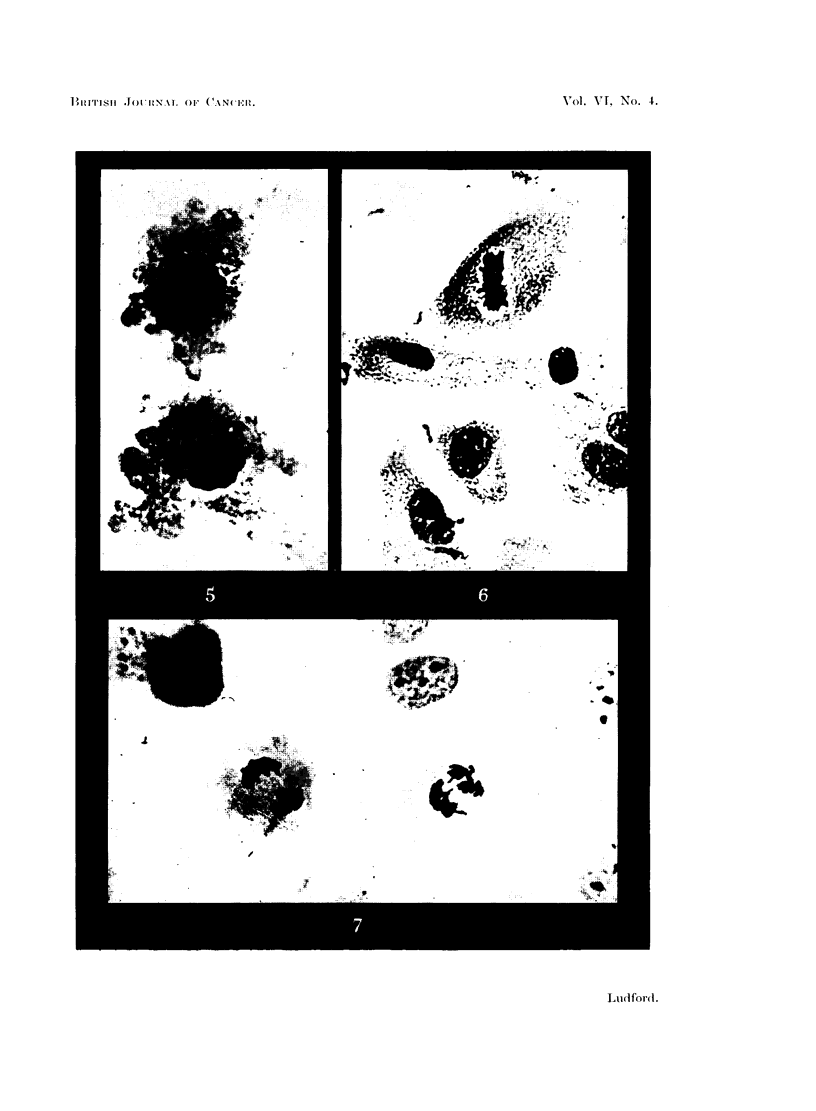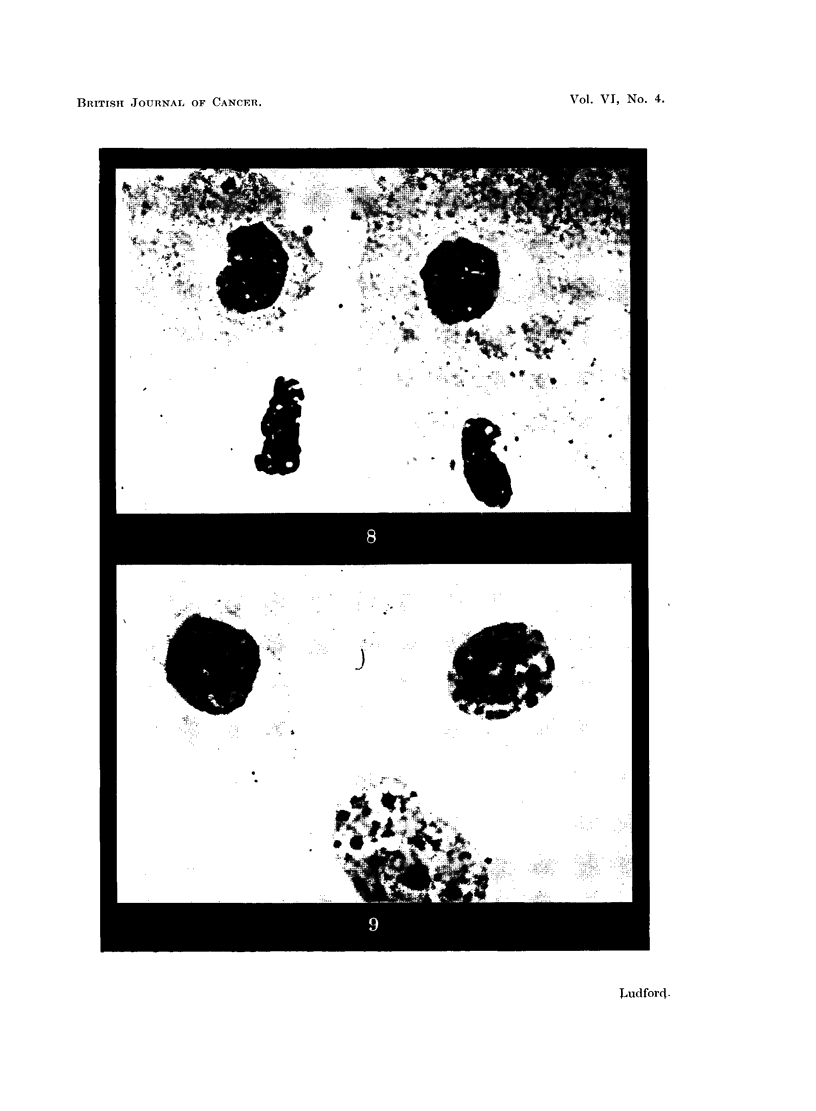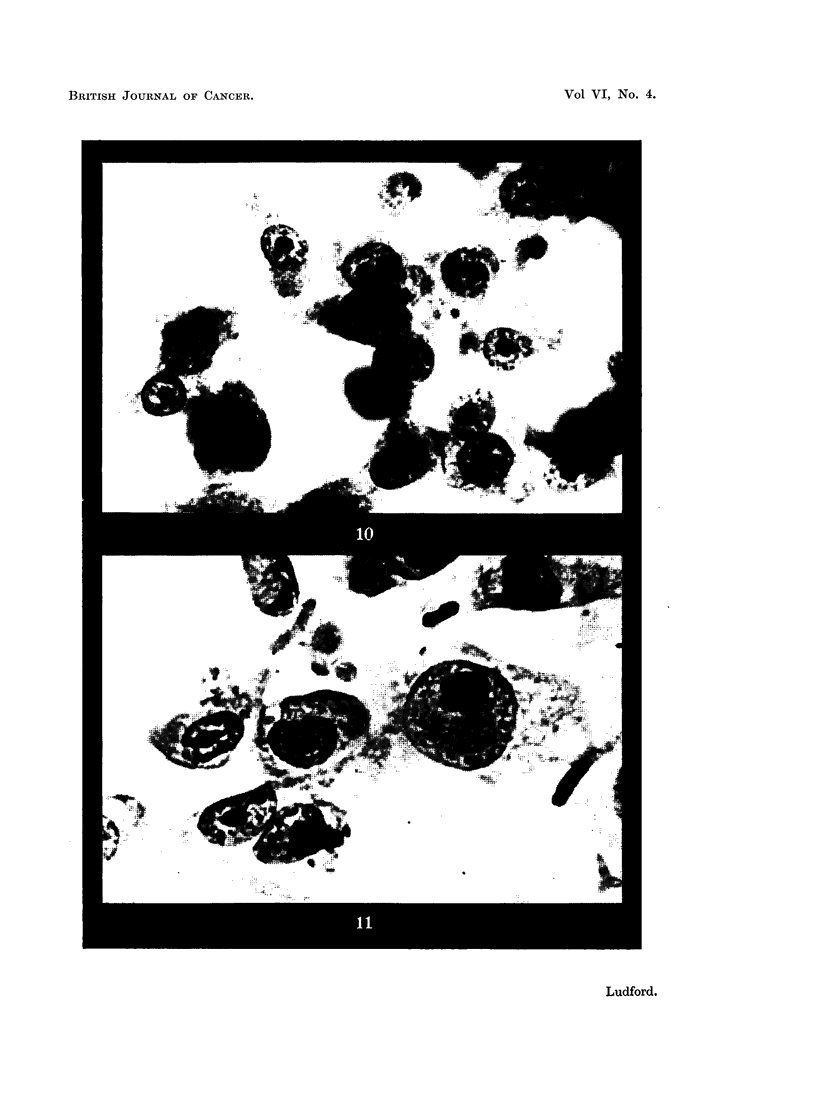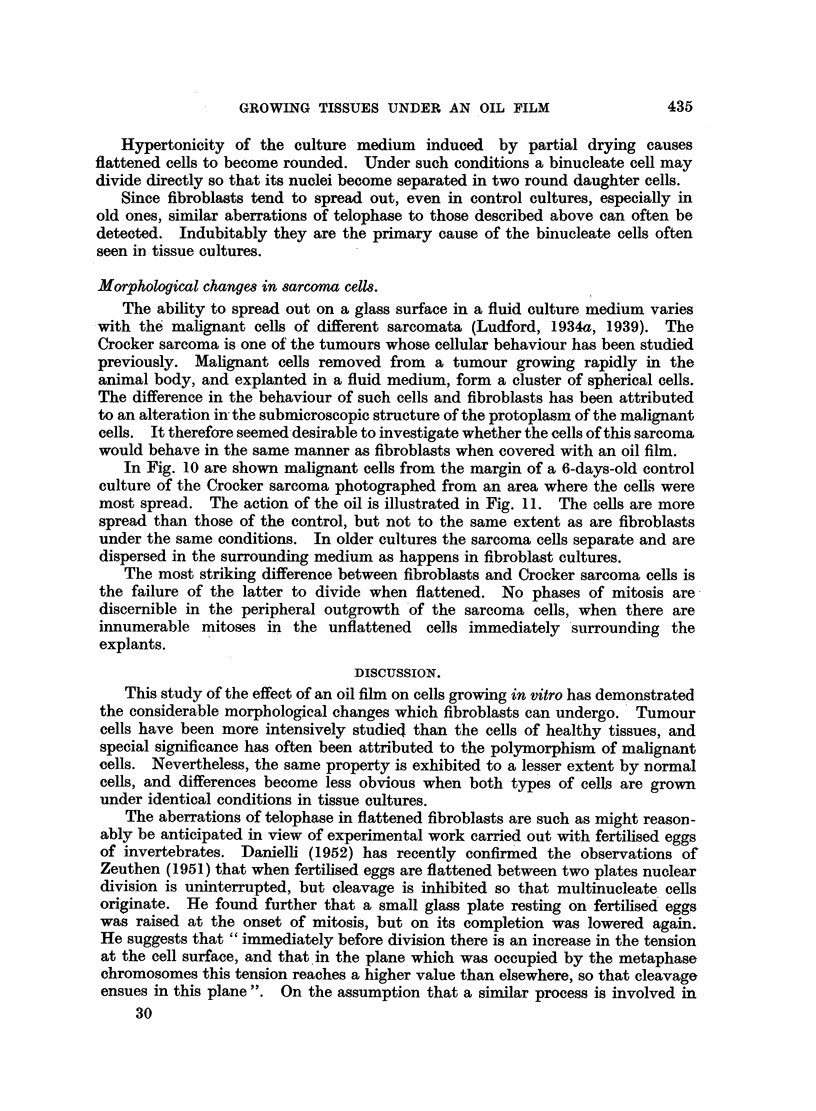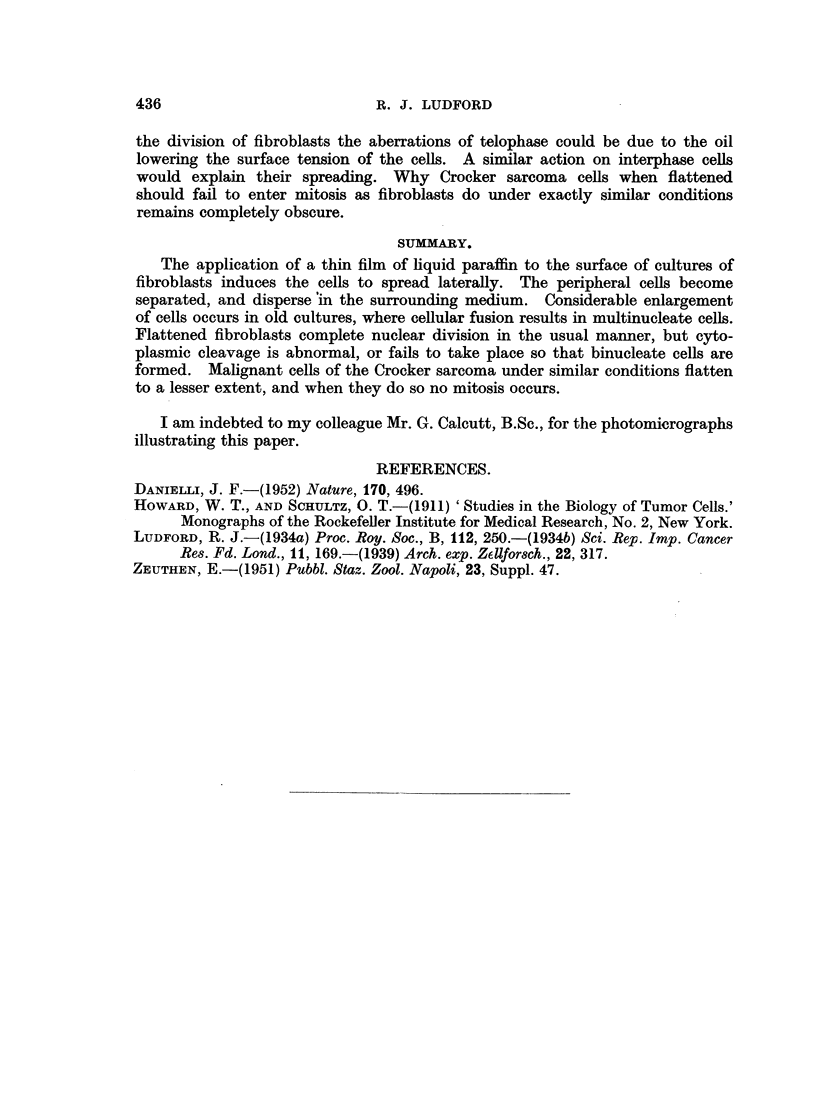# Cytological Results of Growing Tissues Under an Oil Film

**DOI:** 10.1038/bjc.1952.49

**Published:** 1952-12

**Authors:** R. J. Ludford

## Abstract

**Images:**


### CYTOLOGICAL RESULTS OF GROWING TISSUES UNDER AN OIL FILM.

R. J. LUDFORD.*

*From Mount Vernon Hospital, Northwood, Middlesex.*

Received for publication November 13, 1952.

FOR investigating the action of fat-soluble substances on living cells *in vitro*, it is usual to apply them, either as suspensoid colloids, or dissolved in the oil phase of emulsions. Ludford (1934*b*) found that fat-soluble coloured compounds, such as Sudan III and Sudan Black, dissolved in serum, and in this form could be used for vitally staining fat droplets in cells. This suggested that another possible way of treating cells with a fat-soluble substance might be to dissolve it in oil and then apply small drops to the surface of explants of cover-slip cultures, for presumably diffusion would then occur into the serum of the culture medium. To investigate this possibility Sudan Black was dissolved in medicinal liquid paraffin, and a small drop was deposited with a syringe on each explant of a number of cultures of fibroblasts and sarcoma cells. In due course fat droplets in all types of cells became coloured. However, certain changes were observed in the cells which continued to grow under the oil films. It was therefore considered desirable that these should be studied before further experimental work was carried out with chemical compounds dissolved in oil.

### EXPERIMENTAL PROCEDURE AND RESULTS.

Cover-slip cultures were used throughout. The culture medium consisted of equal parts of chick embryo or rat spleen extract, and a mixture of fowl plasma and either rat serum or rat plasma. Liquid paraffin was applied to the explants after two or three days' incubation by means of a syringe fitted with a very fine needle. The oil spread over the explants and extended as a thin film over the growing cells. Before fixing cultures they were washed repeatedly with warm Ringer solution. Thorough washing to remove all oil was essential in order to obtain good staining. The fixatives employed included Susa, Alcohol-Bouin and Helly, and staining was carried out with various preparations of carmine and haematoxylin. Observations were confined to primary cultures.

### *Morphological changes in fibroblasts.*

Fibroblasts emerge from explants of mouse embryo heart as elongated spindle-shaped cells (Fig. 1). Twenty-four hours after the application of a drop of liquid paraffin to the surface of a two-, or three-days-old culture the cells are spread out laterally (Fig. 2). With continuation of growth the peripheral cells become separated, and disperse in the surrounding medium (Fig. 3). They flatten until they are little more than fine films of protoplasm. In old cultures some cells

* Campaign Senior Research Fellow.

become greatly enlarged and resemble the giant cells of tumours. Also, areas of cytoplasm are to be seen which contain several nuclei, usually of varying sizes, between which it is impossible to discern any dividing membranes (Fig. 4). Presumably in such instances the fine films of protoplasm, which previously surrounded each nucleus, have coalesced. A comparable fusion of cells occurs in some tumours and has been designated " plasmogamy " (Howard and Schultz, 1911).

FIG. 1.—Fibroblasts from the periphery of a 5-days-old control culture.
FIG. 2.—Part of the periphery of a 3-days-old culture of fibroblasts, 24 hours after covering with liquid paraffin.

Another effect of the oil is to accelerate the outgrowth of fibroblasts from explants, so that after two days well-spread sheets of cells are obtained. Provided that the oil is completely washed off before fixing cultures, their subsequent staining is greatly facilitated because the plasma which has been in contact with the oil does not stain so intensely as that of the controls. Thus it is possible to obtain preparations of extensively spread cells, well stained and without appreciable coloration of the surrounding medium, which obviously facilitates cytological study.

*Mitosis in fibroblasts.*

The flattening of fibroblasts induced by the oil does not prevent nuclear division, but cytoplasmic cleavage is made difficult. Typically, a cell at the onset of mitosis becomes rounded, but this rarely happens in treated cultures. Mitosis occurs throughout the outgrowths, even in isolated peripheral cells.

FIG. 3.—Flattened fibroblasts dispersed around the periphery of a 9-days-old culture, covered with liquid paraffin 6 days.

FIG. 4.—Fusion of enlarged extensively spread fibroblasts from the inner zone of growth of the same culture as depicted in Fig. 3.

Fig. 6 depicts cells on the edge of a culture, and Fig. 7 was photographed from a part of an outgrowth where the cells were separating. It is not uncommon to find two adjacent cells at the same phase of mitosis. This occurrence is illustrated in Fig. 7, where there are two early telophases, and in Fig. 8 where there are two late telophases. The most feasible explanation is that the two cells in each figure are the daughter cells of a preceding division, and that owing to their identical constitution, and the same environmental conditions, they grew synchronously and began to divide at approximately the same time.

Despite the flattened state of the cells the formation of the chromosomes and their separation into two groups occurs in the normal manner. Obviously, the spindle mechanism is not impaired.

The action of the oil is revealed at the end phase of mitosis. Normally, when cleavage begins the peripheral cytoplasm is involved in a " bubbling " process, which continues as the two daughter cells separate. This is illustrated in Fig. 5, which depicts a dividing cell from a control culture photographed at a higher magnification than Fig. 6 and 7. In the cultures treated with oil there are seen various transition stages between cells exhibiting almost the normal amount of "bubbling ", and its complete absence in the most spread cells. The latter fail to divide, and give rise to binucleate cells. Dividing cells with small peripheral bubbles are often seen, and it would seem that complete separation into two daughter cells may occur while the cytoplasm is considerably spread out. Of the flattened cells shown in Fig. 8 the upper one has completed division, except for a connecting strand of protoplasm, but it is doubtful whether the lower one would have done so. The result of nuclear division without cytoplasmic cleavage is seen in Fig. 9. The two nuclei, which have not yet completed the structural organization of the interphase, have drawn apart in the well-spread cytoplasm. Sometimes cells at this phase elongate and although the nuclei are widely separated (Fig. 6), they have not been observed to complete division. Later the two nuclei come together, as in the cell on the right-hand side of the same figure. Occasionally the two nuclei of such cells are seen to enter simultaneously the prophase of another mitosis. Should this be completed the result, of course, will be a cell with double the normal number of chromosomes. To what extent such occurrences rather than endomitosis are responsible for the polyploid cells of cultures is problematical.

---

### DESCRIPTION OF PLATES.

FIG. 5.—Late telophase from a control culture at higher magnification, showing peripheral " bubbling " of the cytoplasm.

FIG. 6.—Mitosis in fibroblasts on the margin of an outgrowth from an explant of mouse embryo heart. Five days' growth : under liquid paraffin 2 days.

FIG. 7.—Two telophases in mouse fibroblasts grown 4 days under liquid paraffin. Six-days-old culture.

FIG. 8 and 9.—Aberrations of the late phases of mitosis in mouse fibroblasts grown under liquid paraffin.

FIG. 8.—Cytoplasmic division almost complete in the top cell; lower cell failing to divide. Five-days-old culture : under liquid paraffin 3 days.

FIG. 9.—Daughter nuclei at late stage of reconstruction, without cytoplasmic cleavage. Six-days-old culture : under liquid paraffin 4 days.

FIG. 10 and 11.—Malignant cells of the Crocker sarcoma.

FIG. 10.—Part of the periphery of a 6-days-old culture where the sarcoma cells are most spread.

FIG. 11.—More extensively spread cells from the margin of a 6-days-old culture covered with liquid paraffin 4 days.

5

6

7

Ludford.

8

9

Ludford.

10

11

Ludford.

Hypertonicity of the culture medium induced by partial drying causes flattened cells to become rounded. Under such conditions a binucleate cell may divide directly so that its nuclei become separated in two round daughter cells.

Since fibroblasts tend to spread out, even in control cultures, especially in old ones, similar aberrations of telophase to those described above can often be detected. Indubitably they are the primary cause of the binucleate cells often seen in tissue cultures.

*Morphological changes in sarcoma cells.*

The ability to spread out on a glass surface in a fluid culture medium varies with the malignant cells of different sarcomata (Ludford, 1934*a*, 1939). The Crocker sarcoma is one of the tumours whose cellular behaviour has been studied previously. Malignant cells removed from a tumour growing rapidly in the animal body, and explanted in a fluid medium, form a cluster of spherical cells. The difference in the behaviour of such cells and fibroblasts has been attributed to an alteration in the submicroscopic structure of the protoplasm of the malignant cells. It therefore seemed desirable to investigate whether the cells of this sarcoma would behave in the same manner as fibroblasts when covered with an oil film.

In Fig. 10 are shown malignant cells from the margin of a 6-days-old control culture of the Crocker sarcoma photographed from an area where the cells were most spread. The action of the oil is illustrated in Fig. 11. The cells are more spread than those of the control, but not to the same extent as are fibroblasts under the same conditions. In older cultures the sarcoma cells separate and are dispersed in the surrounding medium as happens in fibroblast cultures.

The most striking difference between fibroblasts and Crocker sarcoma cells is the failure of the latter to divide when flattened. No phases of mitosis are discernible in the peripheral outgrowth of the sarcoma cells, when there are innumerable mitoses in the unflattened cells immediately surrounding the explants.

### DISCUSSION.

This study of the effect of an oil film on cells growing *in vitro* has demonstrated the considerable morphological changes which fibroblasts can undergo. Tumour cells have been more intensively studied than the cells of healthy tissues, and special significance has often been attributed to the polymorphism of malignant cells. Nevertheless, the same property is exhibited to a lesser extent by normal cells, and differences become less obvious when both types of cells are grown under identical conditions in tissue cultures.

The aberrations of telophase in flattened fibroblasts are such as might reasonably be anticipated in view of experimental work carried out with fertilised eggs of invertebrates. Danielli (1952) has recently confirmed the observations of Zeuthen (1951) that when fertilised eggs are flattened between two plates nuclear division is uninterrupted, but cleavage is inhibited so that multinucleate cells originate. He found further that a small glass plate resting on fertilised eggs was raised at the onset of mitosis, but on its completion was lowered again. He suggests that " immediately before division there is an increase in the tension at the cell surface, and that in the plane which was occupied by the metaphase chromosomes this tension reaches a higher value than elsewhere, so that cleavage ensues in this plane ". On the assumption that a similar process is involved in the division of fibroblasts the aberrations of telophase could be due to the oil lowering the surface tension of the cells. A similar action on interphase cells would explain their spreading. Why Crocker sarcoma cells when flattened should fail to enter mitosis as fibroblasts do under exactly similar conditions remains completely obscure.

### SUMMARY.

The application of a thin film of liquid paraffin to the surface of cultures of fibroblasts induces the cells to spread laterally. The peripheral cells become separated, and disperse in the surrounding medium. Considerable enlargement of cells occurs in old cultures, where cellular fusion results in multinucleate cells. Flattened fibroblasts complete nuclear division in the usual manner, but cytoplasmic cleavage is abnormal, or fails to take place so that binucleate cells are formed. Malignant cells of the Crocker sarcoma under similar conditions flatten to a lesser extent, and when they do so no mitosis occurs.

I am indebted to my colleague Mr. G. Calcutt, B.Sc., for the photomicrographs illustrating this paper.

### REFERENCES.

DANIELLI, J. F.—(1952) *Nature*, **170**, 496.
HOWARD, W. T., AND SCHULTZ, O. T.—(1911) 'Studies in the Biology of Tumor Cells.' Monographs of the Rockefeller Institute for Medical Research, No. 2, New York.
LUDFORD, R. J.—(1934*a*) *Proc. Roy. Soc.*, B, **112**, 250.—(1934*b*) *Sci. Rep. Imp. Cancer Res. Fd. Lond.*, **11**, 169.—(1939) *Arch. exp. Zellforsch.*, **22**, 317.
ZEUTHEN, E.—(1951) *Pubbl. Staz. Zool. Napoli*, **23**, Suppl. 47.